# Supplementary material for: Deep learning-enabled point-of-care sensing using multiplexed paper-based sensors
Source: NPJ Digit Med. 2020 May 7;3:66. doi: 10.1038/s41746-020-0274-y (PMC7206101; doi:10.1038/s41746-020-0274-y)
Supplement: Supplementary file 1 — Supplementary Information [file 41746_2020_274_MOESM1_ESM.pdf]

Supplementary Materials

DEEP LEARNING-ENABLED POINT-OF-CARE SENSING USING MULTIPLEXED PAPER-BASED SENSORS

Zachary S. Ballard[1,2] , Hyou-Arm Joung[1,3], Artem Goncharov[1], Jesse Liang[2,3], Karina Nugroho[3], Dino Di Carlo[2,3], Omai B. Garner[4], and Aydogan Ozcan[1,2,3,*]

[1] Department of Electrical and Computer Engineering, [2] California NanoSystems Institute,[3] Department of Bioengineering, [4] Department of Pathology and Medicine, University of California, Los Angeles, USA

** Correspondence: ozcan@ucla.edu*

Supplementary Figures

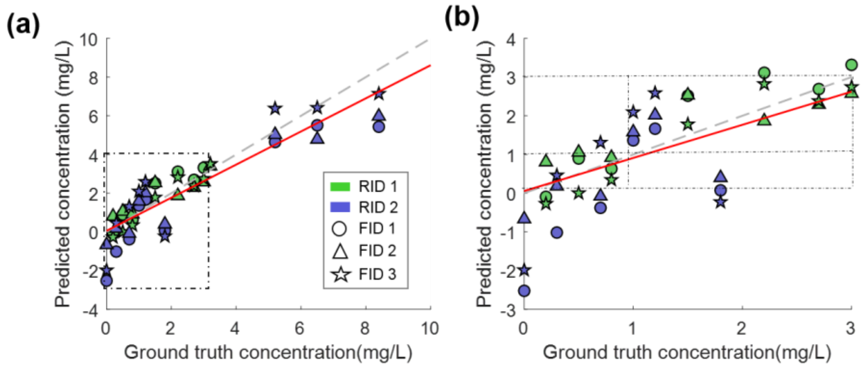

(c)

	Neural Network	Linear Regression
%CV	11.2	47.7
%CV Low	11.5	101.0
%CV Int.	10.1	17.7
%CV High	12.1	11.9
R^2	0.95	0.79

Supplementary Figure 1. Blind testing results of the clinical samples using a multi-variable regression trained on the full x_m inputs (*i.e.* input data defined by all the 81 spots). (shown here for comparison to our approach detailed in the main text). (a) The ground truth CRP concentration plotted against the predicted CRP concentration from blindly tested clinical samples. The marker color and shape represent the different reagent batch ID (RID) and the fabrication batch ID (FID), respectively. (b) The blind testing results for the low and intermediate CVD risk regime, where the dotted lines represent the clinical cut-offs at 1 and 3 mg/L. (c) Table comparing the % coefficient of variation (%CV) and coefficient of determination (R^2) between the neural network based analysis (results show in Figure 3 of the main text) and the multivariable regression, which clearly demonstrate the major advantages of the deep learning based neural net inference.

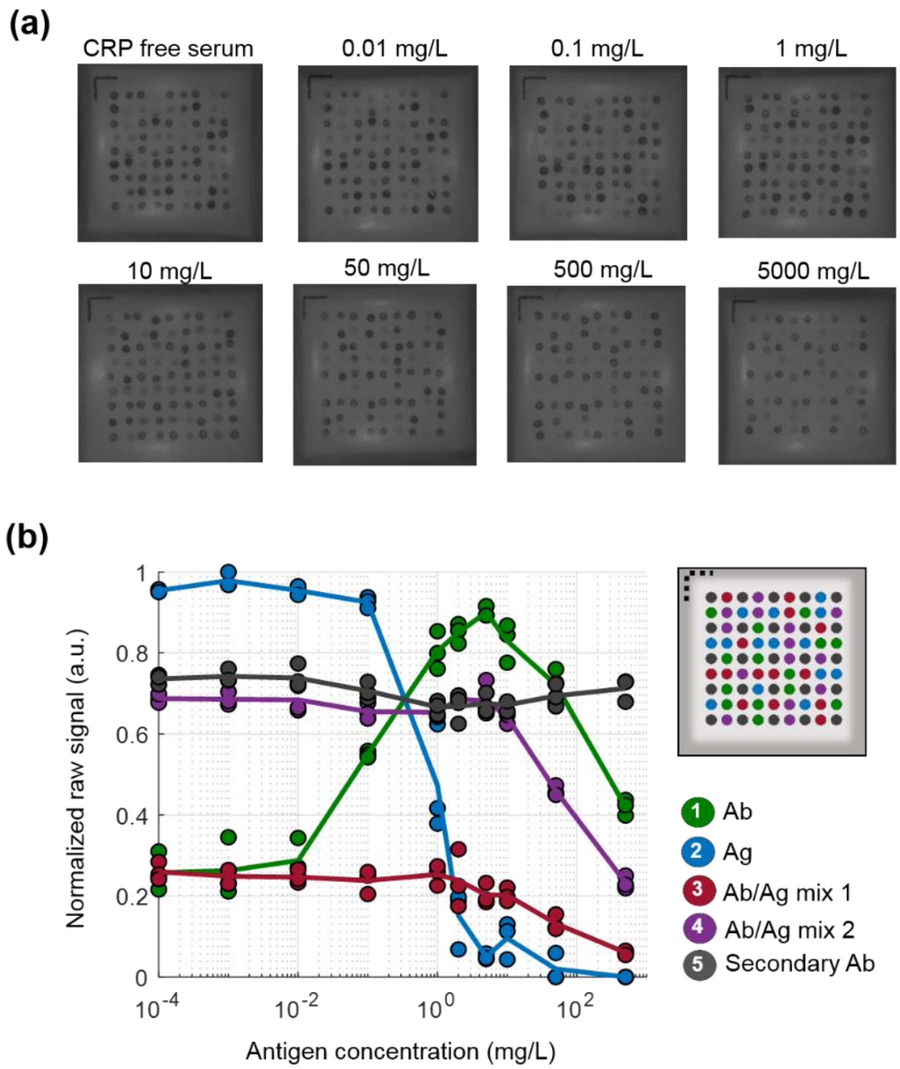

Supplementary Figure 2. Response of the multiplexed sensing channels to different analyte concentrations. (a) Black-and-white images from the mobile-reader of the sensing membranes activated at different CRP concentrations. CRP was spiked into CRP-free serum and run without dilution. The spot-map corresponding to these sensing membranes is shown on the right with a color-coded legend below. (b) The normalized raw signals of five different spotting conditions implemented into the VFA as the sensors are activated with varying CRP concentrations, which were spiked into CRP-free serum. The following spotting conditions were used within PBS buffer: 1) Primary CRP antibody (Ab) at 1 mg/mL; 2) the CRP antigen itself (Ag) at 2.1 mg/mL; 3) A mixture of the CPR Ab and Ag at 0.8 and 0.08 mg/L, respectively; 4) A mixture of the CPR Ab and Ag at 0.8 and 0.24 mg/mL; and 5) the CRP secondary Ab at 0.2 mg/mL.

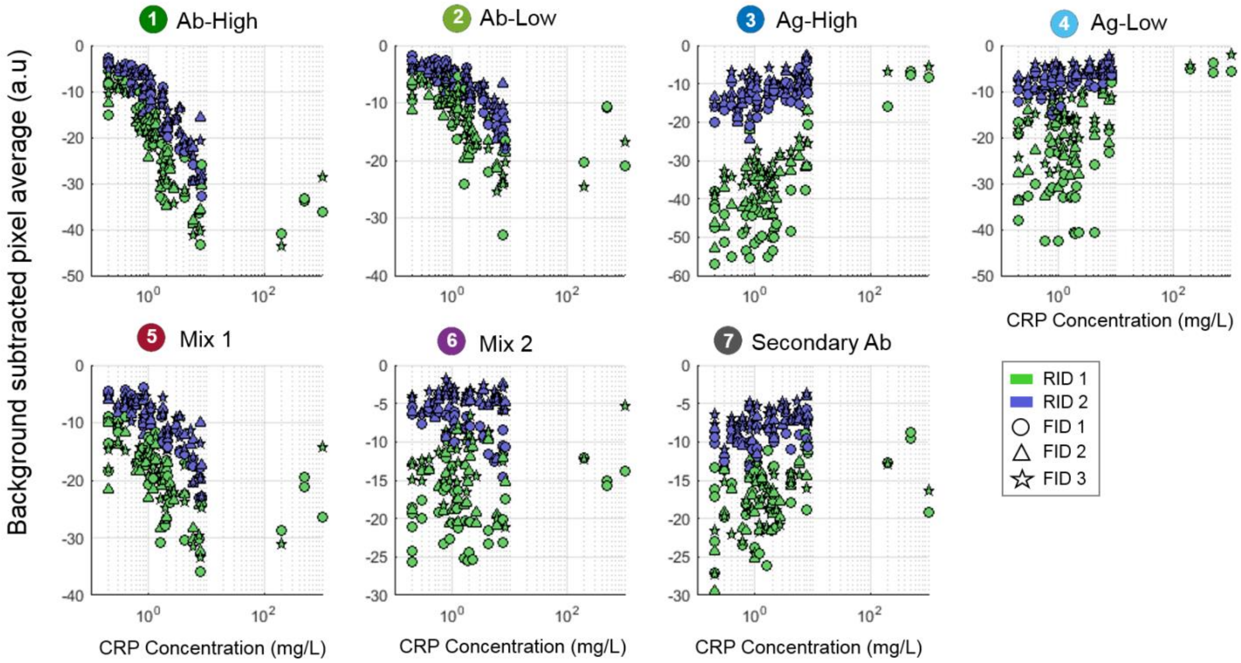

Supplementary Figure 3. Raw data from the training data set of clinical samples. The background-subtracted pixel averages of the immunoreaction spots are plotted against the CRP concentration. Each data point represents the average of like-spots and plotted per spotting condition. The marker color and shape represent the different reagent batch ID (RID) and the fabrication batch ID (FID), respectively.

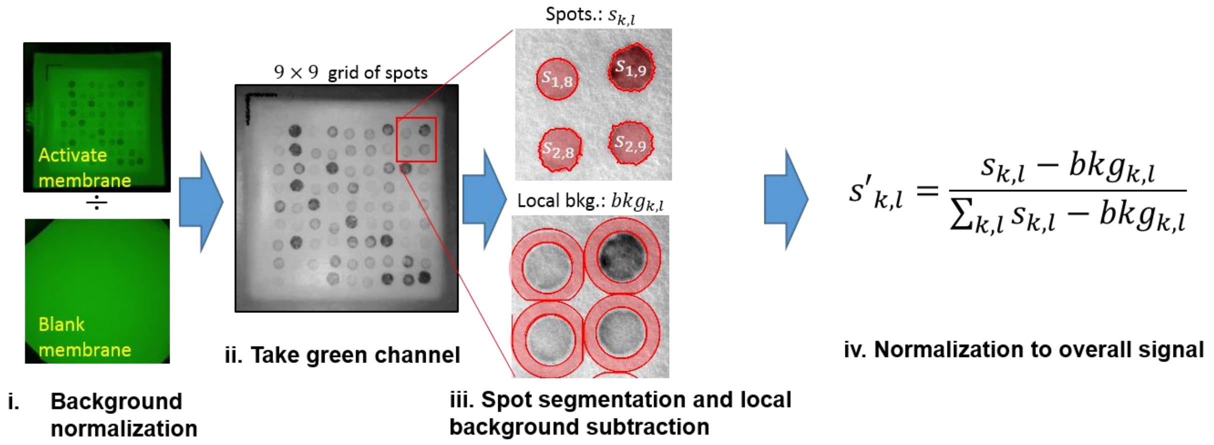

Supplementary Figure 4. Overview of the image processing. i) The image of the sensing membrane is normalized to a universal blank background image (a blank NC membrane), and ii) the green channel is taken. iii) The spots are segmented through an automated algorithm and a local background is taken in a donut-shape outside of the segmented area. iv) The average pixel intensity of the local background $bkg_{k,l}$ is subtracted from the average pixel intensity of the segmented spot $s_{k,l}$ and normalized to the sum of all the background subtracted spot signals. Here, the indices k and l correspond to the row and column locations of the spots on the 9x9 grid, respectively.

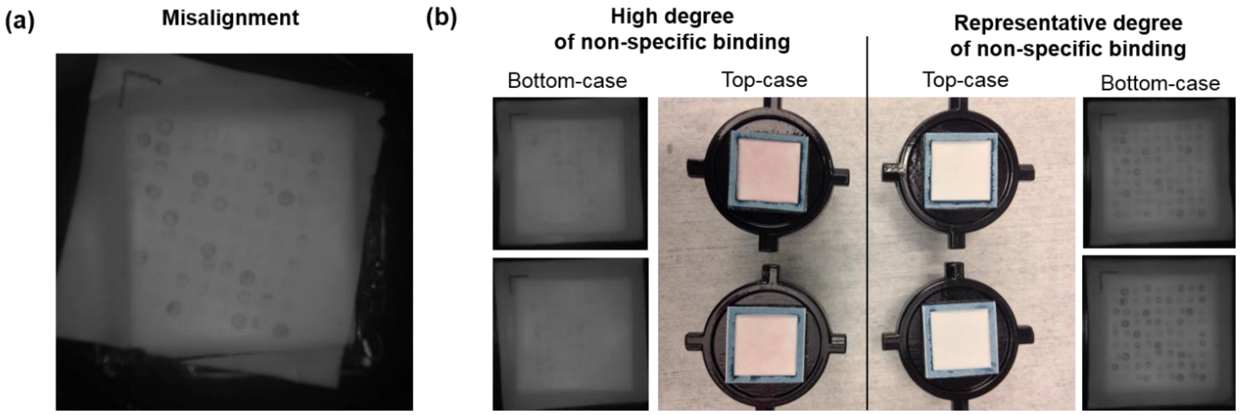

Supplementary Figure 5. Rejected samples from the clinical testing. (a) A misaligned sensing membrane (fabrication error). (b) High non-specific binding in two clinical serum samples which resulted in a pink-color to the paper-layers in the top case and a low overall signal on the sensing membrane (bottom-case). A comparison to a representative top and bottom case for a normal test is shown to the right.

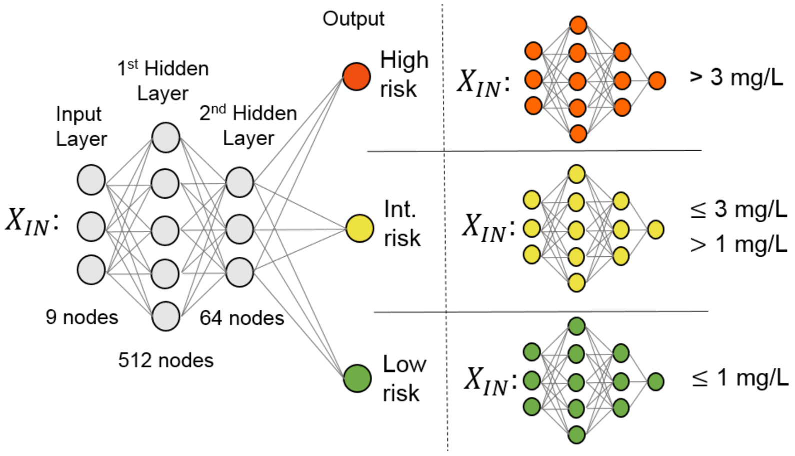

Supplementary Figure 6. The quantification algorithm with a tiered network structure used for cross-validation. The first part of the algorithm (left, in grey) classifies a given sample, defined by the input X_{IN}, into the high, intermediate, or low risk hsCRP regime based off of the clinical cut-offs of 1 and 3 mg/L. The second part of the algorithm then uses separate networks trained with samples within each regime to quantify the CRP concentration of the sample. To avoid edge effects, each quantification network is trained with samples within their cut-off as well as with samples within ±50% of the corresponding cut-off value. Each layer was trained with 50% dropout, ReLu (Rectified Linear Unit) activation function, and a batch size of 22, as determined via a hyper-parameter search. For simplicity, every neural network used the same architecture and hyper-parameters, differing only in the output layer (*i.e.* classification or quantification) and the training data.

Supplementary Tables

Supplementary Table 1. Comparison of point-of-care (POC) xVFA to other high-sensitivity CRP (hsCRP) testing systems. It is important to note that thought these tests (besides the xVFA) are commercially available, there is no FDA approved POC test for hsCRP. A to-scale comparison of the readers and tests is also shown (bottom) along with hyperlinks to the references (Ref) cited in the last column.

	Test Type	Portable?	%CV	R^2	Dynamic Range	Susceptible to Hook-Effect?	Sample Volume	Assay time	Analyzer size	Analyzer weight	Cost (per-Reader, per-Test)	Power Requirement	Ref
**PATHFAST Analyzer (PATHFAST)**	Immunoturbidimetric	No	4.1% (blood)	0.991 (blood)	0.05 – 30 mg/L	Yes	100 µL (serum or blood)	< 17 minutes (serum or blood)	34.3 x 56.9 x 47.5 cm	28 kg	~$17,200, ~$9.75 (retail)	Wall power	[1][2]
**Eurolyser CCA180 (Eurolyzer)**	Immunoturbidimetric	No	5.2% (serum) 8.6% (blood)	0.9995 (serum) 0.9988 (blood)	0.10 - 320 mg/L	Yes	100 µL (serum or blood)	~3 min (serum) ~4 min (blood)	26 x 14.5 x 14 cm	3.4 kg	~$3,299, ~$5.00 (retail)	Wall power	[3] [4]
**QuickSens, hsCRP (Biognostic)**	Colorimetric	Yes	Not Quantitative	Not Quantitative	0.5 – 10 mg/L	Yes	120 µL (serum or blood)	~15 minutes	No Reader	No Reader	No Reader, Not found	None	[5]
**GP 1100 Analyzer (GeTein)**	Immunofluorescent	Somewhat	≤15% (serum)	0.941 (serum)	0.5–200.0 mg/L	Yes	10 µL (serum or blood)	<20 minutes	26.1× 24.1 × 11.5 cm	1.9 kg	$2,300 Not found (retail)	Wall power	[6] [7]
**xVFA**	Colorimetric	Yes	11.2% (serum)	0.950 (serum)	0.1 – 10 mg/L 85 – 1000 mg/L	No	5 µL (serum)	~12 min	15.5 x 8.0 x 6.2 cm	0.36 kg	~$200 ~$1.16 (material cost)	Battery powered	

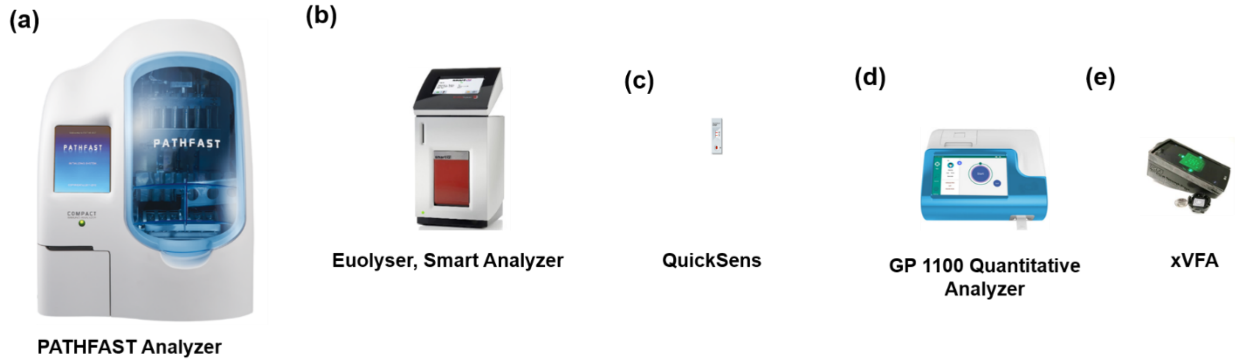

Table Hyperlinks

[1] PATHFAST package insert

[2] PATHFAST retail cost

[3] Eurolyser package insert

[4] Eurolyser retail cost

[5] QuickSens package insert

[6] GP 1100 Analyzer package insert

[7]GP 1100 Analyzer retail cost

Supplementary Table 2. Material specification and cost breakdown of the dry VFA contents. A cross-sectional diagram of the paper layers contained in the VFA is shown on the left with numbers corresponding to the different materials in the table. The multiplexed sensing membrane of the VFA is denoted by the blue dotted outline, contained on the top layer of the bottom case.

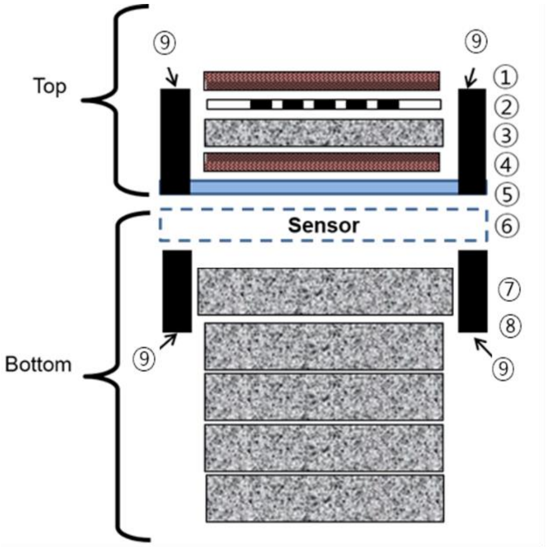

	**Layer**	**Material specification**	**Cost (₵)**
①	Asymmetric membrane	Vivid GX, (1.2x1.2 cm), Pall Co.	0.5
②	Vertical flow diffuser	NC membrane, 0.45 µm pore size (1.2x1.2 cm), Sartorius Stedim North America Inc.	2.9
③	Absorption pad	Whatman grade 707 (1.2x1.2x0.1 cm), Optics Planet, Inc.	1.4
④	Asymmetric membrane	Vivid GX (1.2x1.2 cm) reverse orientation, Pall Co.	0.5
⑤	Supporting membrane	NC membrane, 0.22 µm pore size (1.7x1.7 cm), Sartorius Stedim North America Inc.	2.9
⑥	Multiplexed sensing membrane	NC membrane, 0.22 µm pore size (1.7x1.7cm), Sartorius Stedim North America Inc.	2.9
⑦	Absorption pad	Whatman grade 707 (1.4x1.4x0.18 cm) Optics Planet, Inc.	1.4
⑧	Absorption pad (stack)	Whatman grade 707, (1.2x1.2x1.2x0.18 cm) x 4 pads, Optics Planet, Inc.	5.6
⑨	Foam tape	Foam tape (Super-Cushioning Food-Grade Polyethylene Foam Sheets 1/16"), McMaster-Carr	0.7
		**TOTAL COST**	**18.8₵**

Supplementary Table 3. The seven spotting conditions implemented for the clinical testing with our computational VFA platform (right). The algorithmically determined spot map of the multiplexed sensing membrane (left). Specific colors encode the conditions.

Multiplexed sensing membrane

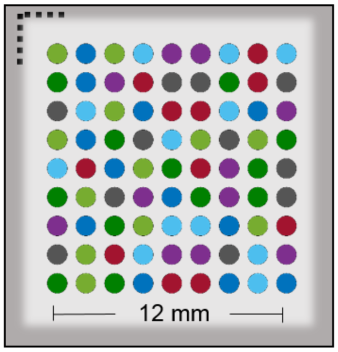

	Condition Abbreviation	Protein	Vendor, Product Info.	Concentration	Cost per spot (¢)	Spots per test
1	Ab-High	CRP Capture Antibody (Ab)	Abcam, ab136176	1 mg/mL	8.9	12
2	Ab-Low	CRP Capture Antibody (Ab)	Abcam, ab136176	0.4 mg/mL	3.6	12
3	Ag- High	CRP Antigen (Ag)	Fitzgerald Industries International, 30-AC05AF	2.1 mg/mL	1.5	12
4	Ag- Low	CRP Antigen (Ag)	Fitzgerald Industries International, 30-AC05AF	1.05 mg/mL	0.8	12
5	Mix 1	Capture Ab + CRP Ag	Combination (see above)	0.8 + 0.08 mg/mL	3.7	11
6	Mix 2	Capture Ab + CRP Ag	Combination (see above)	0.8 + 0.24 mg/mL	3.8	11
7	Secondary Ab	Secondary CRP Antibody	SouthernBiotech, anti-mouse IgG: 1036-01	0.2 mg/mL	0.1	11

Supplementary Methods

The gold nanoparticle-C-Reactive Protein antibody (AuNP-antiCRP) conjugate is synthesized using the following protocol:

1. Mix 900 µl of 40 nm AuNP solution (Ted Pella Inc., 15707-1), 100 µl 0.1M Borate buffer (pH 8.5), and 5 µl anti-CRP mouse IgG antibody (Abcam, ab8278). Incubate the mixture at 25ºC for 1hr.
2. Following the 1-hour incubation, add 100 µl of 1% BSA in PBS solution and mix by vortexing. Then incubate the mixture at 25ºC for 30 minutes.
3. Transfer the mixture to the fridge and incubate at 4℃ for 2 hours.
4. Centrifuge the mixture in a tube at 8000 rpm at 4 ℃ for 15 minutes.
5. After centrifugation, open the tube and discard the supernatant.
6. Add 1 ml of 10 mM tris buffer (pH 7.4) to the microcentrifuge tube containing the AuNP-antCRP mixture and mix by vortexing.
7. Repeat the centrifugation and wash steps (steps 4,5,6) twice to enhance the purity of the mixture.
8. Add 100 µL of storage buffer (0.1 M borate buffers, pH 8.5 with 0.1% BSA and 1% sucrose) to the supernatant and mix via pipetting.
9. Confirm the final concentration of AuNP-antibody conjugates at OD5 through standard optical density measurements at 525 nm.
